# Non-invasive Brain Stimulation and Auditory Verbal Hallucinations: New Techniques and Future Directions

**DOI:** 10.3389/fnins.2015.00515

**Published:** 2016-01-19

**Authors:** Peter Moseley, Ben Alderson-Day, Amanda Ellison, Renaud Jardri, Charles Fernyhough

**Affiliations:** ^1^School of Psychology, University of Central LancashirePreston, UK; ^2^Science Laboratories, Department of Psychology, Durham UniversityDurham, UK; ^3^Centre National de la Recherche Scientifique UMR-9193, SCA-Lab & CHU Lille, Fontan Hospital, CURE Platform, Lille UniversityLille, France

**Keywords:** hallucinations, neurostimulation, neuronavigation, state dependency, transcranial random noise stimulation (tRNS), transcranial direct current stimulation (tDCS), transcranial alternating current stimulation (tACS), transcranial magnetic stimulation (TMS)

## Abstract

Auditory verbal hallucinations (AVHs) are the experience of hearing a voice in the absence of any speaker. Results from recent attempts to treat AVHs with neurostimulation (rTMS or tDCS) to the left temporoparietal junction have not been conclusive, but suggest that it may be a promising treatment option for some individuals. Some evidence suggests that the therapeutic effect of neurostimulation on AVHs may result from modulation of cortical areas involved in the ability to monitor the source of self-generated information. Here, we provide a brief overview of cognitive models and neurostimulation paradigms associated with treatment of AVHs, and discuss techniques that could be explored in the future to improve the efficacy of treatment, including alternating current and random noise stimulation. Technical issues surrounding the use of neurostimulation as a treatment option are discussed (including methods to localize the targeted cortical area, and the state-dependent effects of brain stimulation), as are issues surrounding the acceptability of neurostimulation for adolescent populations and individuals who experience qualitatively different types of AVH.

## Introduction

Auditory verbal hallucinations (AVHs) are the experience of hearing a voice in the absence of any speaker (Aleman and Larøi, 2008). They are commonly associated with a diagnosis of schizophrenia, but also occur in other psychiatric diagnoses such as bipolar disorder and post-traumatic stress disorder (Larøi et al., 2012), as well as in individuals with no psychiatric diagnosis (Beavan et al., 2011; Johns et al., 2014). Evidence from cognitive neuroscience suggests that AVHs are accompanied by high levels of activation in, among other areas, the superior temporal gyrus, particularly in the left hemisphere (Allen et al., 2008; Jardri et al., 2011). Recent attempts to provide novel treatment options for individuals experiencing AVHs have accordingly attempted to use neurostimulation techniques to selectively decrease activity in temporal cortical regions, with a moderate degree of success (Hoffman et al., 2005, 2013; Slotema et al., 2013).

AVHs have been theoretically linked to atypical functioning of inner speech processes, with the most prominent model suggesting that atypical self-monitoring or reality monitoring may lead to a lack of agency over self-generated language processes (Frith, 1992; Jones and Fernyhough, 2007). Evidence from cognitive psychology suggests that individuals with a diagnosis of schizophrenia who experience AVHs, compared to individuals with the same diagnosis who do not experience AVHs, and to healthy controls, are more likely to misattribute self-generated speech in source memory tasks (Stephane et al., 2010) or signal detection tasks (Bentall and Slade, 1985; Brookwell et al., 2013). This is consistent with fMRI research showing that superior temporal cortical regions show high levels of activation both during AVHs (Allen et al., 2008; Jardri et al., 2011) and purposely generated inner speech (Simons et al., 2010). Furthermore, evidence from EEG studies suggests that self- and non-self-generated vocalizations are processed differently in the auditory cortex of healthy, non-hallucinating individuals, as indexed by the N1 event-related potential. This difference was not evident in a sample of individuals with a diagnosis of schizophrenia (Ford et al., 2001; Ford and Mathalon, 2005). These findings have been interpreted as evidence for atypical functioning of forward model mechanisms that usually predict the sensory consequences of self-generated actions. This “efference copy” mechanism acts to attenuate cortical activity in sensory regions resulting from the action, contributing to those actions being experienced as self- or non-self-generated. As such, it has previously been suggested that targeting the left temporoparietal junction (TPJ) or posterior superior temporal gyrus (STG) with neurostimulation may have therapeutic potential because it affects cortical regions involved in the prediction/subsequent sensory attenuation of self-generated actions, such as inner speech (Moseley et al., 2013).

This paper aims to provide a short overview of contemporary research into the efficacy of neurostimulation as a treatment option for AVHs, but also to build upon previous reviews (e.g., Montagne-Larmurier et al., 2011; Moseley et al., 2013) by discussing a number of avenues for future research. In particular, the therapeutic potential of two recently developed techniques, transcranial alternating current stimulation (tACS), and transcranial random noise stimulation (tRNS), are discussed, and it is also suggested that an important line of research may be to maximize efficacy of treatment by utilizing the state dependency of the effects of neurostimulation (i.e., to harness the possibility that neurostimulation may have different effects on cortical excitability levels depending on the state of the brain when it is applied). Furthermore, we discuss a number of technical issues surrounding the use of neurostimulation techniques, such as the most efficient methods for localizing stimulation, and issues surrounding the acceptability and tolerability of neurostimulation in adolescent patients, and for different subtypes of AVH.

## TMS and tDCS as treatment options for auditory verbal hallucinations

Repetitive transcranial magnetic stimulation (rTMS), a non-invasive brain stimulation technique that uses a rapidly changing magnetic field to induce an electrical current in selective cortical regions (Hallett, 2007), has recently shown promise as a treatment option for various neurological disorders such as post-stroke neglect (Cazzoli et al., 2010) or aphasia (Naeser et al., 2010), and psychiatric disorders such as depression (George et al., 1995, 2010). The rationale underlying treatment is that, dependent on the frequency of the repetitive pulses, activity in specific brain regions (or networks of regions) which may be associated with certain disorders can be increased or decreased (Maeda et al., 2000). First tested as a treatment option for AVHs by Hoffman et al. (1999, 2005), low frequency (1 Hz) rTMS over the left temporoparietal junction (TPJ) was employed in a sample of 50 patients with a diagnosis of schizophrenia who hallucinated, in order to reduce cortical activity in this area. Patients received active stimulation or sham stimulation (a control condition in which the participant is led to believe they are receiving TMS, but no stimulation is applied) each day for 15 min, for a total of 9 days, in a parallel design. Using patient-generated narrative reports to create an “Hallucination Change Score” and self-report clinical scales measuring hallucination frequency, vividness, loudness, and attentional salience, it was demonstrated that active rTMS significantly reduced scores, compared to the sham condition. 51.9% of participants in the active condition were classified as “responders” to the treatment (showing a decrease of ≥5 on the Hallucination Change Score), compared to 17.4% in the sham condition.

This initial finding has subsequently been replicated in a number of studies (e.g., Lee et al., 2005; Vercammen et al., 2009; Hoffman et al., 2013), although there are also numerous studies that have not shown a significant effect of low frequency rTMS to the left TPJ on AVH frequency (e.g., McIntosh et al., 2004; de Jesus et al., 2011). Notably, in two of the largest single trials of rTMS efficacy for treating AVHs, Slotema et al. (2011) found no effect of active rTMS, compared to sham rTMS, whilst Koops et al. (2016) found no evidence of efficacy of theta-burst rTMS (consisting of a pattern of pulses thought to have a stronger inhibitory effect) in reduction of AVH frequency, compared to sham. Nevertheless, meta-analyses of studies that have tested therapeutic efficacy of low frequency rTMS on AVHs indicate that it may be effective, with a moderate effect size (Demeulemeester et al., 2012; Slotema et al., 2012, 2013). Given that these meta-analyses suggest an overall effect size of approximately 0.4 on AVH frequency, it is possible that, despite being one of the larger published trials in this area, Slotema et al.'s negative finding may reflect a lack of statistical power.

Studies that have used fMRI to investigate the effects of low frequency rTMS to the left TPJ have shown that a reduction in activity in the left STG is associated with a reduction in AVHs (although there was also a decrease in activity in the left inferior frontal gyrus and anterior cingulate cortex in the active stimulation condition, compared to the sham condition) (Kindler et al., 2013). High levels of activity in the left STG also appears to be a marker for a response to rTMS treatment for AVHs (Homan et al., 2012). Although it seems to be a promising treatment option, further refinement of the technique is needed to establish efficacy; for example, differences in the sham condition and localization techniques used may partially explain inconsistent findings in the literature (see Section Issues with Localization of Targeted Regions).

More recently, transcranial direct current stimulation (tDCS) has also been tested for therapeutic efficacy with AVHs. tDCS involves passing a weak electrical current between two electrodes placed on the scalp, which, dependent on the direction of current flow, depolarizes, or hyperpolarises neuronal membrane potentials. This increases the cortical excitability underneath the anodal electrode and decreases cortical excitability underneath the cathodal electrode (Nitsche and Paulus, 2000; Nitsche et al., 2008). Importantly, the effects of tDCS on cortical excitability may last longer than the period of stimulation, probably mediated by GABAergic and glutamatergic mechanisms (Stagg and Nitsche, 2011). The first use of tDCS as a treatment for AVH was presented in a case report by Homan et al. (2011), in which cathodal stimulation over a posterior STG region was combined with the anodal electrode placed over the right supraorbital cortex. Homan and colleagues reported improvements in hallucination symptoms and reductions in cerebral blood flow in left frontal and temporal regions in a man with persistent, treatment-resistant AVH following 10 days of 1 mA tDCS sessions.

Following this, Brunelin et al. (2012) tested the efficacy of cathodal tDCS (at a strength of 2 mA) to the left TPJ in reducing the frequency of AVHs. Thirty patients with a diagnosis of schizophrenia who hallucinated received tDCS twice a day for five consecutive days, with half receiving active stimulation and half receiving sham stimulation in a parallel design. The cathodal electrode was positioned over the left TPJ, and the anodal electrode over the left dorsolateral prefrontal cortex. Results indicated that active stimulation was associated with a significant decrease in self-reported AVH severity, which was maintained over a 3 month period. There have been comparatively fewer sham-controlled studies utilizing tDCS than rTMS (and therefore not a sufficient number for meta-analysis), and results have been somewhat equivocal, with one study replicating Brunelin et al.'s finding (Mondino et al., 2015) and one study showing no effect of active tDCS, compared to a sham condition (Fitzgerald et al., 2014). If effective, though, tDCS is more tolerable, simpler to apply, and cheaper than rTMS, and so further investigation is needed to test efficacy in larger samples.

The majority of studies testing the efficacy of neurostimulation techniques for AVHs have assessed severity of AVHs in individuals with a diagnosis of schizophrenia or schizoaffective disorder using relatively simple questionnaire measures, most commonly the Auditory Hallucinations Rating Scale (AHRS). The AHRS is a seven-item scale that assesses number of voices experienced, as well as voice frequency, loudness, vividness, attentional salience, length, and distress caused, and has shown acceptable levels of internal consistency, test-retest reliability, and inter-rater reliability (Hoffman et al., 2005). Patients typically complete the AHRS before the first treatment session, after 5–10 sessions of treatment, and, in some studies, up to 3 months later (e.g., Brunelin et al., 2012). Of course, quantifying the success or failure of treatment using this relatively simple measure may exclude observation of other interesting changes that may be of clinical relevance (see Thomas, 2015, for a similar critique of trials of cognitive behavioral therapy for psychosis). As will be argued below, careful attention to phenomenological properties of AVHs will be an important step in fully understanding any therapeutic effect of neurostimulation.

## Alternative neurostimulation techniques

### Transcranial alternating current stimulation (tACS)

The recently-developed technique of tACS uses a sine-wave electric field to affect oscillatory activity in stimulated regions. tACS works on a similar premise to tDCS, by changing the membrane voltages of underlying neurons, hence depolarizing or hyperpolarizing neurons in specific cortical regions. Unlike tDCS, the sine-wave field, theoretically at least, leads to entrainment of a pattern of oscillatory activity at the frequency of stimulation. Research using this technique is still in its early stages, although studies using electroencephalography (EEG) and tACS have suggested that stimulating in the alpha frequency band (8–12 Hz) can lead to enhancement of oscillatory activity at that frequency (Zaehle et al., 2010; Helfrich et al., 2014). Initial research also suggests that frequency-specific stimulation has the potential to affect cognitive task performance. For example, based on previous literature implicating theta frequency oscillations (4–7 Hz) in dorsolateral prefrontal cortex during working memory tasks, Meiron and Lavidor (2014) showed that stimulation at a frequency in the theta band improved performance on an n-back task.

The use of tACS also has the potential for tailoring the frequency of stimulation based on individual oscillatory activity using EEG. This was recently demonstrated by Vosskuhl et al. (2015), who used EEG directly before task performance to determine individual theta frequency, then stimulated at a slightly lower frequency in an attempt to modulate the ratio between theta and gamma (>30 Hz) oscillations in prefrontal cortex. Using this methodology, they showed an improvement in short-term memory performance during stimulation. These studies demonstrate the potential of tACS to affect complex network dynamics by subtly altering ongoing oscillations. It has therefore been suggested that tACS may be a promising therapeutic technique if utilized to alter atypical patterns of oscillatory activity in psychiatric disorders.

Atypical cortical oscillatory activity in the beta (12–30 Hz) and gamma frequencies have been linked to schizophrenia (Uhlhaas and Singer, 2010). Synchronous neural activity is thought to be one way in which disparate neural assemblies communicate and are identified as part of the same functional network (Singer, 1999; Ford et al., 2007a), and as such are likely to play a key role in sensorimotor predictive mechanisms that operate across different brain regions (which, as discussed earlier, are implicated in the genesis of auditory verbal hallucinations). Using EEG, Ford et al. (2007b) have shown lower levels of temporal coherence (a measure of neural synchrony across time) directly before speech in patients with a diagnosis of schizophrenia, which was also associated specifically with the reported severity of auditory hallucinations. Furthermore, in a separate study, Ford et al. (2008) showed that gamma synchrony before a simple motor action was both associated with the size of the subsequent somatosensory event related potential (ERP), and deficient in patients with a diagnosis of schizophrenia. A later study using electrocorticography also indicated that pre-speech gamma synchrony between Broca's area and auditory cortex was associated with the size of subsequent event related potentials (Chen et al., 2011). Attenuation of the N1 ERP has been hypothesized to reflect the functioning of a forward model system which normally predicts the sensory consequences of self-generated actions, and lack of N1 attenuation in response to self-generated actions have previously been associated with schizophrenia (Ford and Mathalon, 2005).

A handful of studies have highlighted either state or trait relations between AVH and neural oscillations. Using symptom-capture measures in which MEG was used whilst participants experienced AVHs, van Lutterveld et al. (2012) observed that a decrease in beta power in the left STG and MTG was associated with hallucination onset (van Lutterveld et al., 2012). An earlier EEG study by Sritharan et al. (2005) also indicated that occurrence of AVH was linked to an increase in alpha coherence (i.e., synchronization) between the left and right auditory cortices. The tendency to experience AVH, meanwhile, has been correlated with auditory steady-state power in the gamma range for left auditory cortex (Spencer et al., 2009) and gamma synchronization between the auditory cortices (Mulert et al., 2011) in people with schizophrenia. As is often the case, however, it is not clear whether differences in power and coherence observed in these studies reflect a cause or effect of the hallucinatory experience.

The literature surrounding the use of tACS as a treatment option for psychiatric disorders is sparse, but Fröhlich et al. (2015) suggest that it should be tested in clinical trials to reduce symptoms known to be associated with atypical oscillatory activity. One avenue of inquiry could be to investigate the therapeutic potential of tACS to entrain or enhance oscillatory activity in patients with auditory verbal hallucinations. For example, stimulating with scalp electrodes placed over inferior frontal and superior temporal areas may be capable of enhancing gamma synchrony between these areas, which, as described above, could improve functioning of forward model systems which ultimately contribute to experiencing (inner) speech as self-generated. Further, comparing the effects of stimulating at different frequencies (i.e., beta and gamma band) could provide information relating to the causal role oscillations in different frequency bands may play in the genesis of AVH.

Drawing on the described research, a number of testable hypotheses can be made regarding the effect of modulating oscillatory activity in patients experiencing AVHs. Firstly, it would be predicted that stimulating frontal and temporal regions in the gamma frequency band would entrain oscillatory activity, decreasing the difference in gamma synchrony between patient and control samples. Secondly, it would be predicted that gamma entrainment would lead to increased sensory attenuation of the N1 ERP in response to self-generated speech. Thirdly, this should be associated with a reduction in the frequency of AVHs.

There are, though, a number of possible issues with using tACS therapeutically. As yet, the length of any after-effects of tACS are unknown. Helfrich et al. (2014) used tACS and EEG simultaneously, showing that stimulation in the alpha frequency range entrained oscillations to the precise frequency of stimulation, but that this effect did not last past the cessation of stimulation. This implies that tACS may not be ideal as a therapeutic tool; however, this study did not use daily stimulation sessions, so it is unclear whether lasting effects would be possible if tACS was used over a 10 day period, as is typical of therapeutic trials using neurostimulation. Future studies should monitor after-effects of tACS using EEG, when used over repeated sessions. This information will be crucial before tACS is tested in a clinical context.

A further question mark over the use of tACS relates to findings indicating that effects may be highly dependent on the state of the brain before stimulation. Feurra et al. (2011) showed that motor cortex excitability (as measured by motor evoked potentials) was increased during beta frequency tACS, inferring that beta oscillations play a causal role in corticospinal excitability. Further work showed that this effect was abolished if the participant was engaged in motor imagery during stimulation; in these conditions, theta frequency stimulation was the only frequency under which motor cortical excitability was increased (Feurra et al., 2013). This is potentially important in the application of tACS to auditory cortical regions, the effects of which could plausibly be modulated by the use of auditory mental imagery and inner speech (both of which may be linked to the occurrence of AVH). The issue of state-dependency is returned to in Section State Dependent Effects of Neurostimulation, below.

### Transcranial random noise stimulation (tRNS)

tRNS is a variant of tACS which also uses a constantly changing current. Whilst tACS stimulates at a set frequency, aiming to entrain oscillatory activity, tRNS stimulates at a randomly changing frequency, usually between 0.1–640 Hz. It has been suggested that tRNS at higher frequencies (>100 Hz) may induce larger excitability changes than stimulating with a direct current (as in tDCS), because the sodium channels of underlying neurons are repeatedly opened by stimulation, and because neuronal homeostatic mechanisms are prevented (i.e., underlying neurons cannot adjust to the constantly randomly changing electrical field; Fertonani et al., 2011). Terney et al. (2008) were the first to demonstrate that tRNS, applied to the motor cortex, increased cortical excitability (as measured by motor evoked potential) and improved performance on a serial reaction time task (associated with implicit motor learning). Fertonani et al. (2011) have also demonstrated that, applied over primary visual cortex, tRNS can improve perceptual learning (as measured by performance on an orientation discrimination task) at a greater rate than tDCS or sham stimulation, whilst tRNS to primary auditory cortex is capable of affecting the auditory steady-state response (Van Doren et al., 2014). Interestingly, Fertonani et al.'s findings suggested a stronger effect when the frequencies were restricted to between 100–640 Hz (compared to < 100 Hz). The authors interpreted this as supporting the argument that a higher rate of repetitive stimulation may lead to a “temporal summation” effect not observable with constant stimulation such as with tDCS (that is, the higher the frequency of stimulation, the more times neurons are stimulated in a short space of time, which may have a summative effect on excitability). Initial findings therefore seem to indicate that tRNS may have a larger effect than tDCS.

As a relatively new technique, there are few reports of therapeutic use of tRNS in neurological and psychiatric disorders. Vanneste et al. (2013) tested the efficacy of tRNS in treating tinnitus, comparing the effects to those of tDCS and tACS over bilateral auditory cortices. The results suggested that tRNS shows promise as a therapeutic technique, yielding larger effect sizes than the other stimulation conditions. Palm et al. (2013) reported a single case in which tRNS with a DC-offset was used over left dorsolateral prefrontal cortex (anode) and right orbitofrontal cortex (cathode) to treat negative symptoms in a 29-year old man with schizophrenia. Moderate improvements were observed in the target symptoms such as emotional withdrawal, along with some amelioration of depression and anxiety. Moreover, the treatment was deemed acceptable and incurred no side effects.

Of more relevance to the treatment of AVHs, in a case study design, Haesebaert et al. (2014) used tRNS offset by 1 mA to test efficacy and safety in the treatment of schizophrenia (including measures of hallucination frequency). The same frontotemporal electrode montage used in previous tDCS studies [see Section Transcranial Alternating Current Stimulation (tACS)] was used, with the anodal electrode placed over left prefrontal cortex and the cathodal electrode placed over the left TPJ. Although only a case study (with no control condition), Haesebaert et al. (2014) showed a decrease in positive and negative symptoms following stimulation, and demonstrated that the technique seems safe and tolerable for the patient. Indeed, two studies have reported that the tactile sensations underneath the electrodes are perceived less with tRNS than with tDCS (Ambrus et al., 2010; Fertonani et al., 2011), suggesting that this may be a preferable technique from the patient's point of view, as well as potentially enabling a more comparable sham condition. Future research should therefore test the efficacy of tRNS applied to TPJ/STG in affecting cognitive mechanisms associated with AVHs, as well as testing its therapeutic efficacy in randomized controlled trials.

## Technical issues

### Issues with localization of targeted regions

Neurostimulation treatment for AVH, applied over the left TPJ, has conventionally used the 10–20 international system, designed for EEG electrode placement, targeting the point midway between the T3 and P3 electrodes. However, one problem with the T3-P3 localization method is that it does not take into account inter-individual anatomical and functional variations, which could be one reason why neurostimulation treatment is not effective for some patients. A more pragmatic approach using an individualized strategy, using neuroimaging data to guide the treatment (neuronavigation), may be able to overcome this issue.

An illustration of how neuronavigation of the TMS coil may lead to therapeutic success in the field of AVH was first provided in a number of case reports. Langguth et al. (2006) used positron emission tomography (PET) with a patient with a diagnosis of schizophrenia, targeting the point of maximal activity in the left temporal cortex with low frequency (1 Hz) rTMS over a number of days, which was followed by a reduction in AVH frequency. Similarly, an fMRI capture of AVH in a child with early-onset schizophrenia (Jardri et al., 2007) and somatosensory hallucinations in an adult schizo-affective patient (Jardri et al., 2008) indicated that neuronavigation may be a useful strategy to localize stimulation.

Later studies have compared groups of hallucinating individuals using either 10–20 based localization methods or neuronavigation methods. In an open-label trial using fMRI whilst patients reported AVHs, rTMS sessions were performed over the individual locations of activation peaks (Sommer et al., 2007). Seven patients received fMRI-guided rTMS, compared to 6 patients treated with T3-P3 rTMS. Although, there was a significant reduction in AVH frequency over the whole sample, no advantage was identified for the neuronavigated group (which may have been linked to the lack of statistical power when comparing small samples). However, a follow-up study with 62 patients, which was split into 3 experimental arms (rTMS targeted at the area of maximal fMRI activity during AVH, rTMS directed at the left TPJ using the 10–20 system, and sham treatment), also found no difference between the localization methods (Slotema et al., 2011). On the contrary, Klirova et al. (2013) reported clinical superiority of neuronavigated rTMS over standard positioning and sham rTMS, although in a smaller sample of 15.

These findings make it difficult to draw conclusions on the effect of fMRI/PET guidance. The general linear model analysis of fMRI used in the described studies has not been shown to provide reliable results at the individual level (Foucher, 2013), and it is possible that the approach used might have been sub-optimal, especially considering recent models suggesting atypical network activity is more important in AVH genesis than any one region (Wolf et al., 2011; Ćurčić-Blake et al., 2015). In a case report providing preliminary evidence for a network approach, Jardri et al. (2009) showed that it may be possible to combine activation maps with fiber bundle tractography between activated functional regions to determine the optimal stimulation target. One of the strengths of this approach is the reference to a functional conceptual framework rather that a “lesional” one; the brain target can be defined as the best point in the network to stimulate, rather than simply expecting a change in activity in one brain region. Indeed, Kindler et al. (2013), using fMRI, showed widespread changes in superior temporal and inferior frontal regions after rTMS treatment, demonstrating wider effects on a network of regions involved in hallucinatory experiences. This functional approach is in accordance with findings regarding the propagation of the effects of rTMS in the entire functional network of a stimulated region (Siebner et al., 2003), and a randomized controlled trial is currently underway to validate such an approach (https://clinicaltrials.gov/ct2/show/NCT01373866).

### State dependent effects of neurostimulation

The efficacy of tDCS, tACS, and tRNS in changing behavioral outcomes has been shown over the last decade to be variable at best, and has recently been criticized as non-replicable (Horvath et al., 2015). Taking tDCS as the most widely used example, the concept of increasing or decreasing cortical excitability via anodal and cathodal stimulation is only truly valid when discussing the motor system, in which the efficacy and mechanism of tDCS was originally elucidated (Nitsche et al., 2008). The reason for this is somewhat obvious; it is easy to examine the excitability and the functional effects of this excitability using subsequent TMS and electromyography recording. There are, however, at least two problems with such a theoretical model being extended to other brain regions: firstly, motor cortex excitability may have no bearing on excitability in other regions of the cortex, particularly secondary cortex (Stewart et al., 2001), and secondly, such a model only takes into account tDCS application to resting state neurons in a neurotypical system.

These issues collectively may explain the heterogeneity in findings relating to anodal and cathodal tDCS effects on behavior in which the anodal/facilitatory, cathodal/inhibitory dichotomy often breaks down (Jacobson et al., 2012). It may be that anodal tDCS is only effective when a task is very familiar (Dockery et al., 2009) or that cathodal tDCS will only negate practice effects but not impair the processing of the task *per se* (Ball et al., 2013). Even in the motor system, voluntary motor contraction can reverse the effects of anodal and cathodal stimulation over M1 (Thirugnanasambandam et al., 2011). As mentioned above, even motor imagery will change the excitability of M1 neurons such that beta frequency tACS no longer facilitates MEPs when imagery is employed, whereas theta frequency tACS will (Feurra et al., 2013). A concurrent combination of excitability increasing events such as fast motor practice and anodal tDCS, which have the same neuronal effect, actually hinders neuroplasticity due to a non-additive mechanism in which the signal-to-noise ratio is already saturated (Bortoletto et al., 2015). In higher level cognition, it is possible to demonstrate a neutralization of the effect of anodal tDCS over left dorsolateral prefrontal cortex in executive function (Gill et al., 2015), and for complex tasks, it is not uncommon to have similar behavioral effects manifested by both anodal and cathodal tDCS albeit via different mechanisms (Miniussi et al., 2013; Knight et al., 2015).

These findings illuminate the partnership that exists between the task demands and its contingent neuronal excitability, and the effect that neurostimulation has on this network. The behavioral consequences of neurostimulation cannot be interpreted without taking these issues into account. This point is critical in the case of tDCS since it is a neuromodulatory technique, and as such can only influence the excitability of neurons, meaning that its effects are dependent on the baseline state of activity. This is in contrast to TMS which will induce action potentials in the underlying tissue (Paulus, 2011; though see below).

In addition to the more transient task related factors already discussed, the state (excitability) of the neurons to be stimulated can be modulated by steady state factors such as age or pathology. There exists recent evidence for the complex interaction between tDCS and the level of excitation in the system which is modulated by the task to affect the final behavioral outcome. Aging has been shown to change the brain both structurally and functionally leading to characteristic changes in behavior (such as visuospatial processing in which pseudoneglect, prevalent in younger adults, disappears in older samples; Benwell et al., 2015). This has been linked to changes in the dominance of hemispheres over the lifespan. However, Learmonth et al. (2015) could find no evidence that age-related changes in excitability modulated tDCS effect. Rather, the effect of tDCS in their lateralized visual detection task was state-dependent in relation to task performance at baseline, with only poor task performers being impaired by anodal tDCS over the left posterior parietal cortex. This would seem to suggest that the task's modulatory effects on neuronal excitability and its interaction with the modulatory effect of tDCS is the key, and adds further context to the contention that baseline performance in addition to practice effects (Dockery et al., 2009; Ball et al., 2013) have a role to play. To further complicate matters, there would seem to be a non-linear interaction between tDCS intensity and baseline performance (Benwell et al., 2015). Further careful work must be done to untangle and further define these relationships, which may be important in clinical applications of tDCS.

The mechanism by which TMS affects the population of neurons under the stimulating coil has also become clearer in recent years. There is now robust evidence from a variety of measures that a TMS pulse will induce an electrical current that will preferentially activate the least active cohort of neurons (Silvanto et al., 2007). First demonstrated using an adaptation paradigm for a variety of visual stimuli, the principle has since been generalized across different stimulation paradigms (suprathreshold and subthreshold TMS, as well as theta burst TMS), different visual areas of the brain, and different paradigms (priming, color, movement, and orientation contingent color) using both psychophysical measures and subjective report (Silvanto et al., 2008).

When considering the use of neurostimulation as a treatment option in AVHs, therefore, the current knowledge concerning factors that may influence their effect across cognitive domains must be integrated. In the case of AVHs, a reduction in activity of left STG is associated with a reduction in frequency (Kindler et al., 2013), perhaps providing evidence that the effect of cathodal tDCS to the left TPJ is consistent with the effects of tDCS applied over motor cortex. However, if applied concurrently with a task that would drive neuronal excitability in one controllable way or another, it may be possible to maximize the clinical effect by defining the underlying state for each patient. Supporting this point, there is evidence that rTMS has greater efficacy in patients with high levels of activity in the left STG pre-treatment (Homan et al., 2012).

It would therefore seem that a further elucidation of these aspects of state dependency of neurostimulation in relation to AVHs would allow us to better tailor a clinical intervention using non-invasive brain stimulation and create a predictive model for its efficacy across the clinical sample.

## Acceptability issues

In general the acceptability and side-effect profile of contemporary neurostimulation techniques for AVH treatment is thought to be good (Sommer et al., 2012), particularly in comparison to use of antipsychotic medication. Although many more trials have been conducted using TMS, single-case reports and group studies suggest that tDCS and tRNS are also acceptable to patients (Homan et al., 2011; Brunelin et al., 2012; Palm et al., 2013).

Nevertheless, the use of neurostimulation techniques will only be appropriate as a treatment option for some people with AVH and not others. The recommendation of neurostimulation (specifically TMS) as a treatment for AVH has been criticized in the past for lacking a strong evidence base, and it has been suggested that such techniques may ignore important psychological and social factors that may be better explored via psychotherapy (e.g., Corstens et al., 2013). Nevertheless, for some people reduction in AVH frequency and persistence will be a specific treatment goal, and neurostimulation may prove to be a viable option.

More broadly, rTMS or tDCS do not appear to be related to any long-term adverse effects if applied within commonly used parameters (relating to frequency, output strength, and stimulation duration in rTMS, and current strength, electrode size, and stimulation duration in tDCS; Brunoni et al., 2012). Hoffman et al. (2005), in a study using rTMS to treat AVHs, reported a higher occurrence of headaches in active rTMS compared to the sham condition, although other adverse effects did not occur more in one condition than the other. Recently developed techniques discussed above (tACS and tRNS) are likely to have similar acceptability to the patient as tDCS, since they use similar equipment. There is some evidence to suggest that the tactile effects of tRNS on the scalp are less perceptible than tDCS (Ambrus et al., 2010; Fertonani et al., 2011), indicating that tRNS may be more tolerable to the participant than tDCS; however, no large-scale study has yet compared the tolerability of tDCS, tACS, and tRNS in a clinical sample. Nevertheless, these three electrical stimulation methods are all likely to have higher tolerability than rTMS, which elicits a larger tactile sensation on the scalp (although is still only mildly uncomfortable for the participant).

### Use in adolescent populations

Neurostimulation may be a promising therapeutic option in adolescent populations because it might help to avoid the adverse developmental consequences of anti-psychotic medication, and frequent suboptimal clinical response (Croarkin et al., 2011). However, in the absence of clear guidelines on the use of non-invasive brain stimulation during developmental periods, the major concern relates to safety. The occurrence of seizures (i.e., one of the most serious TMS-related adverse effects) has been extremely rare in adult populations and none were reported in two large meta-analyses conducted in 850 and 1034 children/adolescents, respectively (Gilbert et al., 2004; Quintana, 2005). TMS-related seizures are more common in high frequency (> 5 Hz) stimulation procedures, whilst treatment of AVHs usually uses low frequency (1 Hz) stimulation (Gilbert, 2008), further lessening the risk of seizure. Furthermore, when observed, transitory neurophysiological changes were not associated with a significant increase in spike-wave discharges in a population of brain-damaged children (Gilbert et al., 2004). rTMS was not found to be associated with cochlear damage or hearing-loss in children or adolescents who received neurostimulation treatment (Collado-Corona et al., 2001). Finally, using a self-report acceptability questionnaire, Garvey et al. (2001) found that for 38 children and adolescents receiving this treatment, the TMS tolerability ranged between a long drive and an appointment to the dentist.

Whilst pilot studies investigating the therapeutic efficacy of rTMS in disorders such as depression, attention-deficit/hyperactivity or autism have been conducted (Croarkin et al., 2011), little is known about the efficacy of rTMS on early-onset AVH. A number of single case-reports have described clinical improvements in the severity of AVH in patients with childhood-onset schizophrenia after low-frequency rTMS (Walter et al., 2001; Fitzgerald et al., 2006; Jardri et al., 2007), and more recently a case-series highlighted the potential beneficial effects of low frequency rTMS on alleviating early-onset refractory hallucinations (Jardri et al., 2012). This case-series provided the first evidence for a significant improvement in the severity of AVH and global functioning after 10 sessions of 1 Hz rTMS over the left TPJ in a group of 10 adolescents with childhood-onset schizophrenia. The therapeutic effect was sustained at the 1-month follow-up and no specific adverse effects were observed. Implementing larger controlled trials is now required to (1) validate 1 Hz rTMS against sham in this population; (2) determine optimized stimulation parameters in developmental periods; and (3) evaluate the long-term duration of the therapeutic effect on early-onset AVH.

### Treatment of subtypes of AVH

There is a growing awareness that AVHs are a heterogeneous phenomenon (Nayani and David, 1996; Jones, 2010; McCarthy-Jones et al., 2014; Woods et al., 2015). Given the variety in underlying cognitive and neural processes likely to be involved in qualitatively distinct AVH subtypes, therapeutic interventions need to be appropriately targeted at relevant underlying processes (Smailes et al., 2015). In this section, we consider the potential applicability of neurostimulation to three common subtypes of AVH: inner speech, memory and hypervigilance hallucinations.

As outlined above, inner speech hallucinations are proposed to arise as a result of a misattribution of an utterance generated in inner speech to an external agent. Targeting selected areas of the fronto-temporal network therefore presents promising opportunities for therapeutic management; as described in Section TMS and tDCS as Treatment Options for Auditory Verbal Hallucinations, it has previously been suggested that stimulating the left TPJ may affect cortical areas involved in self-monitoring, specifically affecting a network of regions involved in inner speech production (Moseley et al., 2013). This is supported by a range of evidence suggesting that superior temporal and temporoparietal regions are involved in discriminating between self- and non-self-generated actions (Blanke et al., 2002; Wang et al., 2011; Moseley et al., 2014) as well-being active during inner speech production (Simons et al., 2010; Alderson-Day and Fernyhough, 2015; Alderson-Day et al., 2016). If the mechanism of action of left TPJ stimulation is indeed via modulation of activity in a self-monitoring network, it is possible that treatment using neurostimulation would be most efficacious for patients experiencing AVHs that are explicable by an inner speech model.

Memory-based AVH are proposed to occur when typical processes of memory retrieval lead to the aberrant generation of an intrusive verbal cognition. The content of such cognitions may relate to the content of what was said during a traumatic event (Jones, 2010). In one model (Waters et al., 2006), the occurrence of the intrusion cognition, coupled with a lack of the contextual information that would usually lead to the cognition being identified as a memory, results in the cognition being attributed to an external source. Existence of memory-based AVHs is supported by cluster analysis of data relating to phenomenological properties of AVHs (McCarthy-Jones et al., 2012) indicating that these AVHs may be distinct from those related to inner speech.

It is unclear whether neurostimulation would be an effective therapeutic option for memory-based AVHs. Evidence from fMRI has shown that some AVHs may be preceded by activation in left parahippocampal regions (Diederen et al., 2010), which the authors interpret as evidence that areas involved in memory recollection may dysfunctionally trigger language related regions, resulting in AVH. Although subcortical regions such as parahippocampal cortex cannot easily be targeted by transcranial techniques such as rTMS or tDCS, their interaction with temporal language regions may be affected by stimulation of the left TPJ. Furthermore, stimulation of prefrontal regions (as is common in tDCS montages) may be able to modulate top-down control involved in metacognitive processes, which may relate to the intrusiveness with which resurfacing memories are experienced (Jones and Fernyhough, 2006; Fleming and Dolan, 2012). Some evidence for this comes from the literature on post-traumatic stress disorder, in which a number of studies have shown efficacy of high frequency (20 Hz) rTMS to the left or right dorsolateral prefrontal cortex in the reduction of PTSD symptoms (Boggio et al., 2010). Interestingly, a recent paper has suggested that some AVHs associated with schizophrenia and with PTSD may share common phenomenological and aetiological mechanisms (McCarthy-Jones and Longden, 2015). Nevertheless, a more in-depth understanding of memory-based AVH, both phenomenologically and at a neural level, is required before confident predictions can be made about neurostimulation efficacy.

A third subtype of AVH, termed hypervigilance AVH (Dodgson and Gordon, 2009) may differ in its cognitive and neural substrates from both inner speech and memory-based AVH. These are defined as the perception of a threat-related utterance in the context of a noisy environment. Hypervigilance AVH appear to result from top-down biases toward the perception of certain emotionally salient stimuli, and have recently been accounted for within a predictive processing framework (Wilkinson, 2014). Although, little is known about the neural basis of hypervigilance AVH, it might be predicted that cortical areas involved in attentional biases, particularly in the auditory modality, would be involved in these AVHs. An extensive body of research using dichotic listening paradigms has linked AVHs to biased attentional processes (Hugdahl et al., 2008, 2012), with some evidence suggesting that interhemispheric synchrony (between left and right auditory cortices) may be atypical in individuals that hallucinate (Mulert et al., 2011; Steinmann et al., 2014). This may be related to the ability to exert cognitive control over attentional processes, which could feasibly be related to hypervigilance AVH. If so, neurostimulation may be best targeted to normalize activity in bilateral auditory cortical regions (using anodal and cathodal stimulation), or to enhance neural synchrony between these regions using gamma-frequency tACS. Alternatively, it is possible that these AVHs may be more amenable to psychological therapies which aim to alter patient's appraisal of the experiences (Smailes et al., 2015).

Overall, a deeper understanding of the cognitive and neural mechanisms of different subtypes of AVH is needed before confident predictions can be made about neurostimulation efficacy. To date, neuroimaging studies of AVHs simply tend to compare hallucinating and non-hallucinating patients (usually with a diagnosis of schizophrenia) with healthy controls, but a more fruitful approach may be to divide samples into groups based on phenomenological variables relating to inner speech, memory, and hypervigilance processes. In this way, treatment options could be targeted with higher success rates, and in particular, treatment using techniques such as rTMS, tDCS, tRNS, or tACS might be applied to different regions, dependent on the likelihood of efficacy. It is likely that the heterogeneity of current findings regarding efficacy of neurostimulation treatment is, at least partially, because some types of AVH are more likely than others to be affected by stimulation of the left TPJ.

## Summary

Here, we have outlined a number of future avenues for research into the use of neurostimulation techniques as a treatment option for AVHs. To summarize, whilst studies testing the efficacy of rTMS and tDCS indicate that they may be effective at reducing AVH frequency, new techniques such as tACS and tRNS should be tested, both in clinical trials and in relation to their effect on self-monitoring and inner speech processes in healthy populations. This paper has argued that, due to it's effects on cortical oscillatory activity, tACS may be capable of affecting network communication between frontal and temporal regions, thought to be involved in predictive models which relate to self-monitoring. tRNS, meanwhile, may be a more effective option than tDCS, potentially over-riding homeostatic mechanisms that may lessen the effect of tDCS on excitability.

There are also outstanding questions relating to the best approaches to localizing the target of stimulation. The evidence so far does not strongly support efficacy of neuronavigated rTMS compared to the T3-P3 method, but further research taking into account more complex inter-individual differences in structural and functional connectivity may increase efficacy. An important future direction for research will also be to explore the best way to harness state dependent effects of neurostimulation, which may have the potential to further increase the effectiveness of treatment. There are also issues relating to acceptability and utility in adolescent samples, or individuals experiencing qualitatively different types of AVH, which will be important to address in future research.

## Funding

This work was supported by the Wellcome Trust (grant numbers: WT098455MA & WT108720).

### Conflict of interest statement

The authors declare that the research was conducted in the absence of any commercial or financial relationships that could be construed as a potential conflict of interest.
